# Acute TBK1/IKK-ε Inhibition Enhances the Generation of Disease-Associated Microglia-Like Phenotype Upon Cortical Stab-Wound Injury

**DOI:** 10.3389/fnagi.2021.684171

**Published:** 2021-07-13

**Authors:** Rida Rehman, Lilla Tar, Adeyemi Jubril Olamide, Zhenghui Li, Jan Kassubek, Tobias Böckers, Jochen Weishaupt, Albert Ludolph, Diana Wiesner, Francesco Roselli

**Affiliations:** ^1^Department of Neurology, Ulm University, Ulm, Germany; ^2^German Center for Neurodegenerative Diseases (DZNE)-Ulm, Ulm, Germany; ^3^Master in Translational and Molecular Neuroscience, Ulm University, Ulm, Germany; ^4^Department of Neurosurgery, Kaifeng Central Hospital, Kaifeng, China; ^5^Institute of Anatomy and Cell Biology, Ulm University, Ulm, Germany; ^6^Neurozentrum Ulm, Ulm, Germany

**Keywords:** TBK1, microglia, traumatic brain injury, amlexanox, neuroinflammation, stab wound injury

## Abstract

Traumatic brain injury has a poorer prognosis in elderly patients, possibly because of the enhanced inflammatory response characteristic of advanced age, known as “inflammaging.” Recently, reduced activation of the TANK-Binding-Kinase 1 (Tbk1) pathway has been linked to age-associated neurodegeneration and neuroinflammation. Here we investigated how the blockade of Tbk1 and of the closely related IKK-ε by the small molecule Amlexanox could modify the microglial and immune response to cortical stab-wound injury in mice. We demonstrated that Tbk1/IKK-ε inhibition resulted in a massive expansion of microglial cells characterized by the TMEM119^+^/CD11c^+^ phenotype, expressing high levels of CD68 and CD317, and with the upregulation of Cst7a, Prgn and Ccl4 and the decrease in the expression levels of Tmem119 itself and P2yr12, thus a profile close to Disease-Associated Microglia (DAM, a subset of reactive microglia abundant in Alzheimer’s Disease and other neurodegenerative conditions). Furthermore, Tbk1/IKK-ε inhibition increased the infiltration of CD3^+^ lymphocytes, CD169^+^ macrophages and CD11c^+^/CD169^+^ cells. The enhanced immune response was associated with increased expression of Il-33, Ifn-g, Il-17, and Il-19. This upsurge in the response to the stab wound was associated with the expanded astroglial scars and increased deposition of chondroitin-sulfate proteoglycans at 7 days post injury. Thus, Tbk1/IKK-ε blockade results in a massive expansion of microglial cells with a phenotype resembling DAM and with the substantial enhancement of neuroinflammatory responses. In this context, the induction of DAM is associated with a detrimental outcome such as larger injury-related glial scars. Thus, the Tbk1/IKK-ε pathway is critical to repress neuroinflammation upon stab-wound injury and Tbk1/IKK-ε inhibitors may provide an innovative approach to investigate the consequences of DAM induction.

## Introduction

Traumatic Brain Injury (TBI) is a major worldwide cause of morbidity and mortality (Bruns and Hauser, 2003; Jiang et al., 2019). The incidence of TBI is not homogeneously distributed throughout ages. At least, two peaks have been recognized in epidemiological datasets, the first attributed to the adolescent-young adult population (15–24 years) and the second occurring in the geriatric population (Kraus et al., 1984; Tiret et al., 1990; Bruns and Hauser, 2003; Peeters et al., 2015). These two subgroups are often conflated when clinical trials are performed, under the assumption that, although age may be a risk factor for TBI, the unfolding of TBI-associated cascades may be similar. This notion is challenged by the worse prognosis observed in older patients with TBI (Thompson et al., 2006) and by experimental evidence of distinct microglial phenotypes and heightened neuroinflammation in aged mice (Ritzel et al., 2019; Early et al., 2020).

Aging *per se* is characterized by an enhanced inflammatory response at systemic (Salminen et al., 2012; Franceschi and Campisi, 2014; Mogilenko et al., 2021) as well as at brain level (Cheng H. et al., 2018; Mejias et al., 2018). According to the “inflammaging” concept, a low-grade inflammatory state is characteristic of advanced age and this primed inflammatory response contributes to a heightened response upon stimuli. Notably, microglial transcriptomes are substantially different in the advanced age (Galatro et al., 2017; Hammond et al., 2019) and recently a microglial phenotype resembling Disease-Associated Microglia (DAM) has been identified in the white matter upon aging (Keren-Shaul et al., 2017; Raj et al., 2017; Safaiyan et al., 2021), where it appears to be involved in the disposal of degenerated myelin. Interestingly, DAM also characterizes several age-related neurodegenerative conditions, such as Alzheimer Disease and Amyotrophic Lateral Sclerosis (Ofengeim et al., 2017; Dols-Icardo et al., 2020; Mifflin et al., 2021) and it is characterized by a distinctive transcriptional profile including the upregulation of *Cst7, Clec7a, CSFR1*, and *ApoE* genes (Krasemann et al., 2017; Keren-Shaul et al., 2017; Rangaraju et al., 2018) and it is believed to be relevant for the phagocytosis of apoptotic cells and for the formation of amyloid plaques (Anderson et al., 2019; Muth et al., 2019; Srinivasan et al., 2019; Sobue et al., 2021). Additional aging-associated subpopulations of microglia have been reported, in particular interferon-driven and inflammatory subpopulations (Hammond et al., 2019) which appear to be related to cognitive impairment (Deczkowska et al., 2018). Irrespective of subpopulation phenotypes, microglia from aging brains secretes higher levels of IL-6 and IL1b (Rawji et al., 2016), expresses a higher level of Toll Like Receptor (TLR) and Major Histocompatibility Complex (MHC) proteins (Letiembre et al., 2009), but display reduced motility and impaired phagocytic capacity (Koellhoffer et al., 2017; Gabandé-Rodríguez et al., 2020). In line with these findings, older mice display a more abundant microglial proliferation and peripheral leukocytes infiltration (in particular neutrophils) upon TBI, larger production of reactive oxygen species, and higher levels of TNF-alpha secretion (Ritzel et al., 2019).

The investigation of the molecular mechanisms involved in the age-related enhanced microglial reactivity has recently identified the TANK1-binding kinase 1 (Tbk1) a player in the inflammaging phenotype. In Tbk1 ± mice, the transcriptional profile characteristic of age-associated pro-inflammatory state appears earlier and progresses faster than in wild-type littermates (Bruno et al., 2020), without overt neurodegeneration. Likewise, blood-brain-barrier permeability is disrupted in Tbk1 ± mice (Alami et al., 2020) and conditional myeloid loss of Tbk1 results in a pro-inflammatory state and cellular infiltrate in the spinal cord and other organs (Duan et al., 2021). Moreover, loss of Tbk1 has been shown to synergize with TAK1 downregulation in bringing about the age-associated activation of Receptor-interacting serine/threonine-protein kinase-1 (RIPK1) and related inflammaging phenotype (Xu et al., 2018). Thus, chronic decrease in Tbk1 activity is hypothesized to be associated with increased inflammatory responses.

The anti-inflammatory agent Amlexanox (AMX; Reilly et al., 2013) is an inhibitor of Tbk1 and of the closely related IKK-ε (Cheng H. et al., 2018). AMX has been shown to reduce canonical signaling through NF-kB (Cheng C. et al., 2018; Möser et al., 2019) by inhibiting IKK-ε and Tbk1, while, through inhibition of Tbk1, causing the de-repression of non-canonical signaling through NIK/NF-kB (Jin et al., 2012). Furthermore, AMX-induced inhibition of Tbk1 has been linked to the decrease in phosphorylation of IRF3 and IRF7 (Mori et al., 2015; Quan et al., 2019; Zhou et al., 2020), implying the downregulation of Tbk1-mediated Interferon signaling. AMX was originally approved for human use in dermatological conditions (Abbasi et al., 2016), and, because of its effects on reducing NF-kB and IRF3/7 activation (through the double targeting of Tbk1 and IKK-ε) AMX has been proposed as a therapeutic agent in autoimmune neuroinflammatory disorders (Quan et al., 2019), neoplastic progression (Wilcz-Villega et al., 2020) and diabetes-associated macrophage-driven inflammation (Oral et al., 2017).

Here, we have investigated the effect of acute Tbk1/IKK-ε blockade by AMX administration in a model of focal traumatic (stab wound) brain injury. We conceptualized two possible outcomes: a predominant anti-inflammatory effect, as seen in the context of other inflammatory conditions, or a predominant pro-inflammatory effect, in agreement to the enhanced reactivity observed upon Tbk1 deletion and in aging. We found that AMX treatment caused the increase in microglial and lymphocytic infiltration in the injury site, associated with the upregulation of a subset of inflammatory cytokines such as IL-17 and IFN-gamma, and that, most notably, microglial cells assumed a phenotype similar to DAM (Keren-Shaul et al., 2017). AMX treatment was ultimately found to lead to a larger astrocytic scar after injury.

## Materials and Methods

### Animals

Male mice originated from the breeding of wild-type male and female B6SJL/F1 mice (from Jackson labs^1^) were used for the present study at the age of 55–65 days. Mice were maintained at 22°C with a 12/12 h light/dark cycle and had food and water *ad libitum* as previously reported (Alami et al., 2020). The present study has been authorized by Ulm University Animal Experimentation oversight service and by the Regierungspräsidium Tübingen under the permit no. 1379; the study has been performed in agreement with the national animal welfare legislation. In order to minimize confounding factors deriving from oestrus cycle and fluctuating levels of hormones, we employed only male mice for this study.

### Stab Wound Injury

The stab wound injury (SWI) was performed as previously reported (Frik et al., 2018; Wiesner et al., 2018). Briefly, 60 days old mice were anesthetized with an intraperitoneal injection of midazolam, medetomidine, and fentanyl (5 mg/kg; 0.5 mg/kg; 0.05 mg/kg). A unilateral craniotomy was performed 1 mm above bregma and 1 mm ventral the cranial sutures (x = + 1.0, y = + 1.0). The blade was inserted into the primary motor cortex up to a depth of 0.8 mm (*z* = −0.8) and moved 1 mm in the dorsal direction. Three parallel stab-injuries were performed with a gap of 0.2 mm; this gap was chosen so that the three injuries would actually form a single lesion area. Afterward the craniotomy was covered and anesthesia was antagonized with atipamezole-flumazenil-buprenorphine-injection (2.5 mg/kg; 0.5 mg/kg; 0.1 mg/kg). Animals were monitored daily for the appearance of motor impairment, but no mouse displayed disturbances requiring humane euthanization. The health status of the animals was monitored twice daily after the procedure and until the end of the experiment. If any animal had shown severe weight loss (>20%), motor impairment, hunched posture, apathy or other signs of permanent impairment related to the procedure, it would have been removed from the experiment and killed painlessly; no animal was actually euthanized because of discomfort. Starting from the day of injury, mice were treated with 100 mg/kg Amlexanox or vehicle (5% PEG400; 5% Tween20; 90% NaCl 0.9%) administered by oral gavage for 7 consecutive days, once daily. The first treatment with Amlexanox (AMX) or vehicle by oral gavage was given 2 h after surgery and then daily (every 24 h). Mice were either sacrificed 7 or 40 days after the trauma.

For the biochemical analysis, animals were sacrificed by cervical dislocation, the brain was extracted and 1.5 mm-diameter cortex biopsies from the injured region (or from the control primary motor cortex) were obtained, sealed in 1.5 ml eppendorf tubes, and frozen in liquid nitrogen. For histological analysis, animals were deeply anesthetized with 1 mg/kg body weight ketamine chlorhydrate and 0.5 mg/kg body weight xylazine, and transcardially perfused with 1.5 ml/g of ice-cold PBS and then with 1.5 ml/g of 4% paraformaldehyde in 0.1M pH 7.4 phosphate buffer. Brains were thereafter dissected out, post-fixed for 24 h in 4% paraformaldehyde, and cryoprotected for 48 h with 30% sucrose in PBS before being embedded in Optimum Cutting Temperature (OCT) sectioning medium. Upon cryostat sectioning (40 μm thickness), sections were collected from 0.75 mm caudally to the bregma until 1.75 mm rostrally to the bregma, spanning the injury site. Only sections corresponding to the middle part of the injury site were considered, once those with obvious artifacts had been discarded.

### Real Time (RT)- Quantitative Polymerase Chain Reaction (qPCR)

RNA was isolated from the ipsilateral and contralateral cortical samples using the ISOLATE II RNA/DNA/Protein Kit (Bioline) according to the manufacturer’s instruction. Reverse transcription was performed with 0.75μg RNA using reverse transcriptase (Promega), RNase Inhibitor (RiboLock, Thermo Scientific), dNTPs (Genaxxon), and random hexamers (Biomers). qPCR was performed on the LightCycler 480II (Roche) with the Power PCR SYBR green PCR master mix (Takara). 2 μl of cDNA was used in a total volume of 10 μl (3 μl primer mix and 5 μl of SYBR green) in a 96-well plate. The following parameters were used for the amplification: Holding stage :95°C (30 s).

$$\mathrm{Cycling}\mathrm{stage}\left(40\mathrm{cyles}\right):95^{{}^{\circ}}C\left(5s\right)\to 60^{{}^{\circ}}C\left(20s\right);$$

$$\mathrm{melt}\mathrm{curve}\mathrm{stage}:95^{{}^{\circ}}C\left(10s\right)\to 60^{{}^{\circ}}C\left(60s\right);$$

clooldown:hold 4°C. Samples were duplicated and the housekeeping gene GAPDH was used as a control (for the cytokine primer sequences see Supplementary Table 1). Data were analyzed using the Cycler software and normalized to the normalization factor according to the following equation: 2-ΔCt (ΔCt = Cttarget gene—CtGapdh) = relative mRNA.

### Western Blotting

Protein lysates were prepared using Radioimmunoprecipitation assay (RIPA) buffer (150 mM NaCl, 10 mM Tris pH 7,6, 0,1% SDS, 1% Triton X-100, 5 mM EDTA). Protease (Roche) and phosphatase inhibitors (Sigma Aldrich) were added to the buffer. Protein concentrations in the samples were determined using the BiCinchoninic Acid (BCA) assay kit (Thermo Fisher Scientific). The Protein samples (30 μg) were then run on an 10% gel for 2 h at 60V and transferred to a nitrocellulose membrane using semidry blotting method (Ouali-Alami et al., 2020). The membranes were blocked for 1 h at room temperature with 5% skim milk powder or 5% BSA dissolved in Tris-Buffered Saline-Tween (TBS-T), followed by an overnight incubation of primary antibodies at 4°C: rabbit anti NAK/TBK1, 1:2,000 (Abcam); rabbit anti NAK/TBK1 (Ser 172), 1:2,000 (Abcam); mouse anti-β-Actin, 1:5,000 (CST); rabbit anti SQSTM1/p62, 1:2,000 (Abcam); rabbit anti phospho-SQSTM1/p62 (Ser403), 1:1,000 (GeneTex); rabbit anti STING, 1:1,000 (Cell signaling); rabbit anti phospho-STING (Ser365), 1:1,000 (Cell signaling), mouse anti PSD95, 1:1,200 (abcam) rabbit anti NR1, 1:1,000 (Sigma). The blots were then incubated in a secondary goat conjugated IgG-HRP antibody (rabbit, mouse, or rat; depending on the primary host) for 1 h at RT. The blots were visualized (Enhanced chemiluminescence; ECL-immunodetection) using BIORAD ImageLab (duration of exposure time 1–20 s). Samples were corrected for background and quantified using BIORAD Image Lab Software^®^ 5.0, following manufacturer’s procedures. All values were normalized first to the housekeeping genes (β-actin) and then to their respective total protein. The list of material used has been summarized in Supplementary Table 2.

### Immunohistochemistry

Free-floating sections were blocked for 2 h at RT in 3% BSA and 0.3% Triton in Phosphate-buffered saline (PBS) and incubated with primary antibodies [rabbit anti-TMEM119; 1:100 (Abcam), mouse anti-CD11c; 1:100 (Abcam), rat anti-CD169; 1:200 (BioLegend) rabbit anti-CD3; 1:100 (Abcam), mouse anti-CD45; 1:500 (BD biosciences), mouse anti-CD68; 1:500 (Abcam), mouse anti-CD317; 1:100 (R&D Systems), mouse anti-CS-56; 1:200 (Abcam), rat anti-GFAP; 1:200 (Thermo Fisher Scientific)], mouse anti-NeuN; 1:100 (Millipore), for 48 h at 4°C. Sections were washed for 3 × 30 min in PBS and incubated for 2 h at RT with secondary antibodies [Donkey anti-mouse 568, 1:500 (Invitrogen); Donkey anti-rat 488, 1:500 (Invitrogen); Donkey anti-rabbit 568, 1:500 (Invitrogen)]; Donkey anti-mouse 647, 1:1,000 (Invitrogen) together with DAPI (1:1,000). After a second round of washing (3 × 30 min in PBS), sections were mounted with Fluorogold prolong antifade mounting medium (Invitrogen). The list of material used has been summarized in Supplementary Table 2.

### Image Analysis

The images (2 × 2 tile) were acquired in 1024 × 1024 pixel 12-bit format, using a Leica DMi8 microscope, equipped with an ACS APO 40x oil objective. Imaging parameters were set to obtain signals from the stained antibody while avoiding saturation. To avoid fluorescence cross-bleed, all fluorescent channels were acquired independently. All experiments included 3 animals per group with 4 brain sections each, sampling the stab injury site, were imaged. Confocal stacks (20 optical sections each) were collapsed in maximum intensity projection pictures. For quantification of TMEM119^+^, CD11c^+^, CD3^+^, CD45^+^, CD68^+^, CD317^+^, CD169^+^, and NeuN^+^ cells, images were subjected to thresholding (based on the histogram of the images, with a value cutting off the lower 20% of pixels) with the sole purpose of objectively identifying “positive” from “negative” cells, and a region of interest of 2 × 10^4^ μm^2^ located at the injury site was selected. The ROI was positioned on the axis of the injury stab at a constant distance from the pial surface, chosen so that the ROI is located within layer II–III.

In the region of interest, the absolute number of cells (each cell identified by the presence of a DAPI-positive nucleus) was manually counted and recorded for statistical analysis; we defined as “cell density” the number of cells per area unit. For quantification of CS-56 and GFAP images were subjected to thresholding but, in contrast to the counts of microglial cells, the cumulative positive area above the threshold was calculated (this considering the scar area as the ultimate readout, irrespective of the number of astrocytes composing it).

### Statistical Procedures

All datasets were tested for normality by the Shapiro-Wilk test. Outliers were screened by the ROUT algorythm with Q set at 1%. No outlier was identified or eliminated during the data analysis. For statistical analysis, one-way ANOVA was used for comparing mean values that were considered significantly different. For analysis of TMEM 119, CD169, and CD11c cells, one-way ANOVA was used when the factors treatment (vehicle, AMX) vs. injury (sham, TBI) were compared. Tukey’s *post hoc* comparison was applied to take into account the multiple comparisons. Data are presented either as the standard error of the mean (SEM) or mean standard deviation (SD). Graph Prism software (GraphPad Software Inc.) was used to perform statistical analysis. Detailed statistics for each experiment are reported in Supplementary Tables 2–10. The evaluation of the images and of expression data was performed by two experiments (RR and LT), whereas the treatment was performed by an independent experiment (DW). The experiments involved in the analysis were blind to the treatment group but were not blind to the presence/absence of injury, since the lesion is obvious in the microscopy images.

## Results

### Amlexanox Inhibits the Phosphorylation of Tbk1 Substrates in the Brain

**FIGURE 1:**
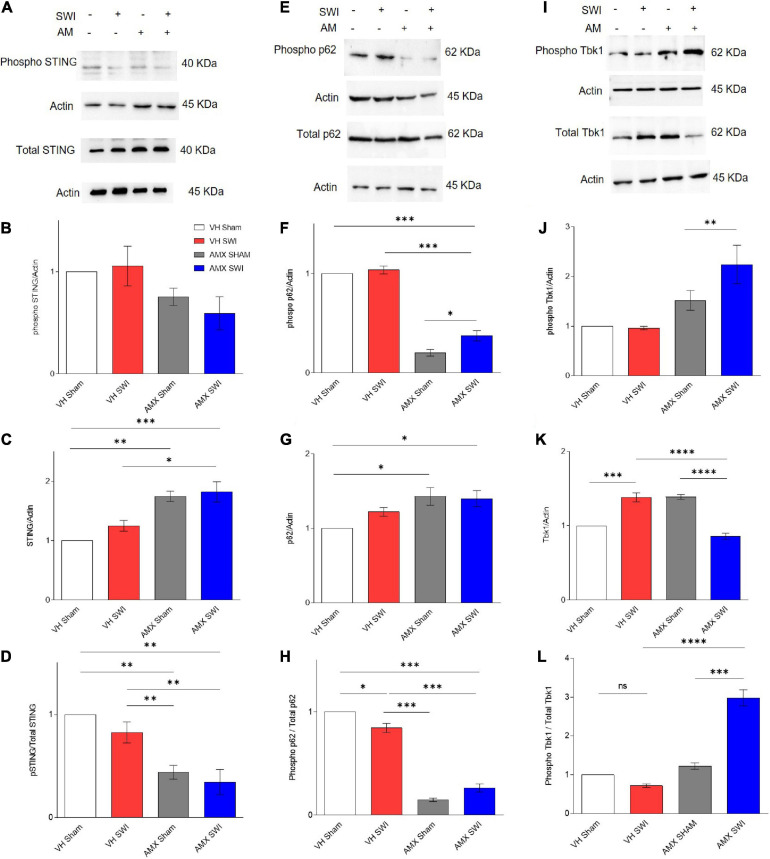
Amlexanox (AMX) reduces the phosphorylation of Tbk1 targets STING and p62 upon Stab Wound Injury (SWI). Western blots of cortical biopsies (at 7 dpi) homogenates show trend toward decreased levels of phospho STING **(A,B)** but an increase in total STING levels **(A,C)**, resulting in a significant decrease in the fraction of phosphorylated STING **(D)**. Likewise, levels of phospho p62 **(E,F)** were significantly decreased the AMX treatment (irrespective of SWI) whereas the total amount of p62 was slightly increased **(E,G)**, resulting in a substantial decrease in the fraction of phosphorylated p62 in the overall p62 pool **(H)**. Levels of phosphorylated Tbk1 were strongly increased in AMX-SWI samples **(I,J)** whereas total levels of Tbk1 were reduced in these samples (but slightly increased by AMX or SWI alone) **(K)**, resulting in a massive increase in the fraction of phosphorylated Tbk1 in the Tbk1 pool in AMX-SWI samples **(L)**. *n* = 3 animals/group. Average ± SEM; one-way ANOVA ns: not significant, ^∗^*p* < 0.05, ^∗∗^*p* < 0.01, ^∗∗∗^*p* < 0.001, ^****^p < 0.0001; full statistical report in Supplementary Table 3; original full uncropped WB in Supplementary File 1.

We set out to demonstrate target engagement by AMX, by verifying that downstream substrates of Tbk1/IKK-ε were down-phosphorylated upon inhibitor treatment (Figure 1). Tbk1/IKK-ε sport a very large set of substrates (Kim et al., 2013), many of which relevant to inflammation and aging. We consider only a small subset of them to confirm that AMX was penetrating the brain parenchyma in concentrations sufficient to significantly block Tbk1/IKK-ε activity. First, we considered the total levels of Stimulator of Interferon Genes (STING) protein and of STING phosphorylated on Ser366 (pSTING), a site specifically targeted by Tbk1 (Liu et al., 2015). pSTING (S366) showed a trend toward reduced levels in AMX treated mice (subject to sham surgery or Stab Wound Injury; SWI) (Figures 1A,B). Importantly, total levels of STING were substantially increased by the AMX treatment (irrespective of the injury status; Figures 1A,C; VH Sham vs. AMX Sham: *p* = 0.0017, VH Sham vs. AMX SWI: *p* = 0.0008, VH SWI vs. AMX Sham: *p* = 0.0293, VH SWI vs. AMX SWI: *p* = 0.0122), in agreement with the role of Tbk1 in stimulating STING degradation (Prabakaran et al., 2018). Overall, the fraction of phosphorylated STING was decreased to less than 50% of baseline (vehicle-treated, sham surgery mice) upon AMX treatment (Figure 1D; VH Sham vs. AMX Sham: *p* = 0.0028, VH SWI vs. AMX SWI: *p* = 0.0090). Second, we monitored the total levels of p62 and the levels of p62 phosphorylated on S403 (phospho-p62), which is another typical phosphorylation target of Tbk1 (Matsumoto et al., 2015). We observed in AMX treated mice a significant decrease in phospho-p62 levels (Figures 1E,F) while observing at the same an increase in total p62 levels (Figures 1E,G), corresponding to the substantial decrease in the fraction of the total pool of p62 phosphorylated on S403, confirming the effective blockade of p62 phosphorylation and its degradation upon AMX treatment (Figures 1E,H; VH Sham vs. VH SWI: *p* = 0.0178, VH Sham vs. AMX Sham: *p* < 0.0001, VH Sham vs. AMX SWI: *p* < 0.0001, VH SWI vs. AMX Sham: *p* < 0.0001, VH SWI vs. AMX SWI: *p* < 0.0001).

Levels of phosphorylated Tbk1 were not upregulated upon SWI in vehicle-treated mice (Figures 1I,J) but were significantly increased by AMX treatment, and even further in AMX-SWI samples (Figures 1I,J). Interesting, total Tbk1 levels were upregulated by SWI in vehicle-treated mice as well as in AMX-treated sham animals, but were downregulated in SWI-AMX samples (Figures 1I,K; VH Sham vs. VH SWI: *p* = 0.0006, VH Sham vs. AMX Sham: *p* = 0.0006, VH SWI vs. AMX SWI: *p* < 0.0001, AMX Sham vs. AMX SWI: *p* < 0.0001). However, the ratio of phospho Tbk1/total Tbk1 was actually increased in presence of AMX (indicating that a larger fraction of Tbk1 was actually phosphorylated despite the decrease in total protein level) and in particular in AMX-SWI samples (Figures 1I,L; VH SWI vs. AMX Sham: *p* < 0.0001, VH SWI vs. AMX SWI: *p* < 0.0001, AMX SHAM vs. AMX SWI: *p* < 0.0001), suggesting that even if the autophosphorylation ability of Tbk1 dimers is suppressed, other upstream kinases can still phosphorylate Tbk1 (Clark et al., 2009) and that although reduced in total quantity, remaining Tbk1 is heavily phosphorylated. Taken together, these data show that a substantial inhibition of the Tbk1 pathway takes place in the brain upon systemic AMX treatment, indicating a substantial penetration of the brain by the drug and providing proof of target engagement.

### Tbk1 Inhibition Drives the Massive Expansion of CD11c^+^ Disease-Associated Microglia and the Infiltration of Peripheral Immune Cells Upon SWI

**FIGURE 2:**
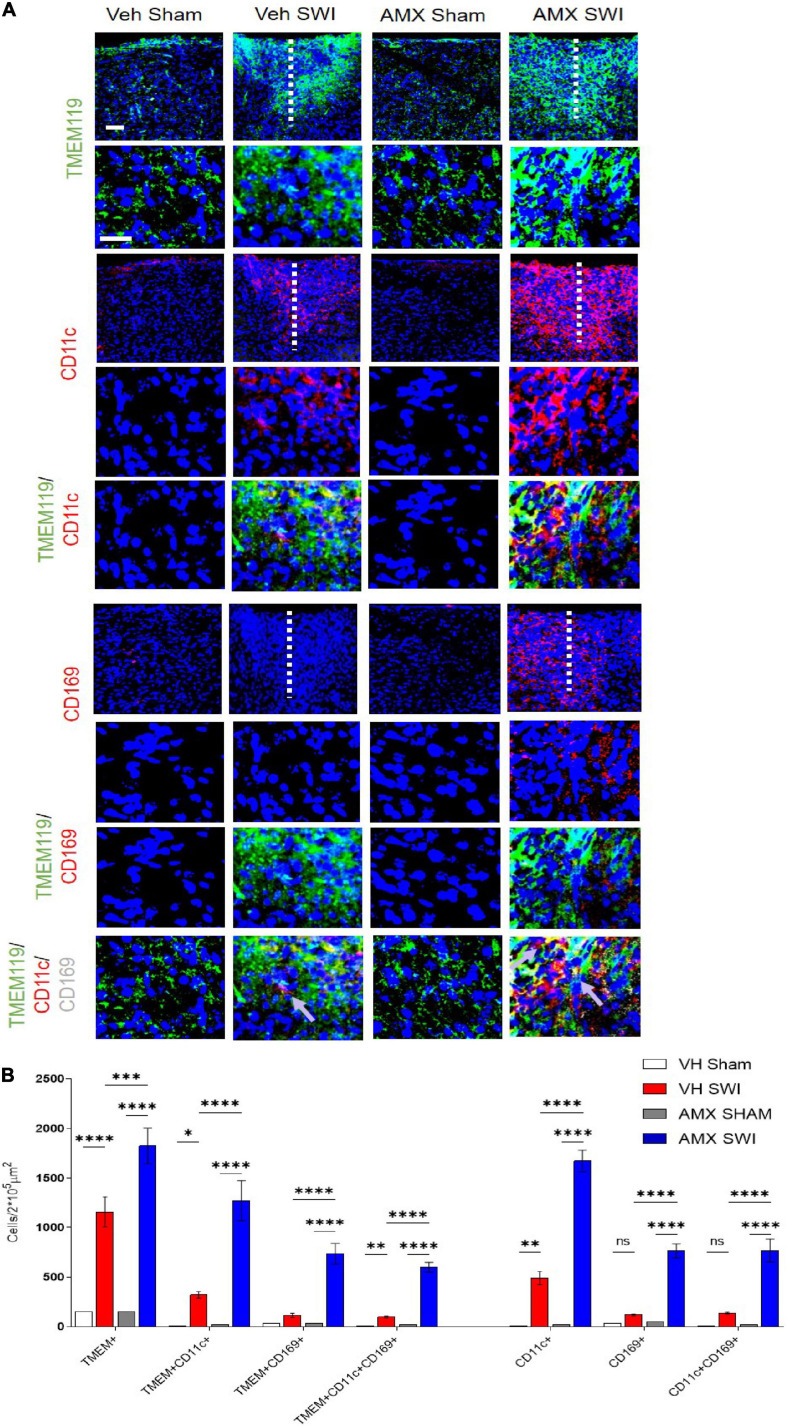
Tbk1 blockade enhances the induction of CD11c^+^ and CD169^+^ microglia upon Stab Wound Injury (SWI). **(A,B)** AMX treatment caused the significant elevation in TMEM119^+^ microglial cells in the SWI injury site at 7 dpi. Co-immunostaining with makers of microglial subsets CD11c and CD169 revealed the massive expansion of CD11c^+^ microglia (disease associated microglia; DAM) as well as reactive CD11c^+^/CD169^+^ microglia. (*n* = 3 animals/group with 4 sections/animal. Average ± SD; one-way ANOVA with Tukey’s post hoc; ns: not significant, ^∗^*p* < 0.05, ^∗∗^*p* < 0.01, ^∗∗∗^*p* < 0.001, ^****^p < 0.0001; full statistical analysis in Supplementary Table 4). Upper panel, low magnification; lower panel, high magnification. Scale bars (equal for both sets of panels) 200 μm.

Next, we set out to evaluate the effect of Tbk1/IKK-ε inhibition by AMX on the microglial response triggered by SW injury (SWI). This injury model was chosen because of its very high reproducibility in terms of injury severity and location, and because of its established role for the study of microglial and astroglial responses to injury. Mice subject to SWI were treated with either vehicle or AMX (100 mg/kg) daily and were sacrificed at 7 dpi. Microglial cells were identified by TMEM119 immunostaining (Bennett et al., 2016). We also took into consideration two subpopulations of microglia: TMEM 119^+^/CD11c^+^ cells (compatible with disease-associated microglia, DAM; Keren-Shaul et al., 2017) and TMEM119^+^/CD169^+^ cells (reactive microglia; Bogie et al., 2018). In sham-operated animals, the density of microglial cells and their subpopulations were comparable in vehicle- and AMX-treated mice. When compared to sham mice, SWI caused in vehicle-treated mice a strong increase in the number of TMEM119^+^ microglial cells in the region surrounding the injury site. Remarkably, in SWI mice treated with AMX, the microglial density was substantially larger than in vehicle-treated mice (Figure 2; VH Sham vs. VH SWI, VH Sham vs. AMX SWI, AMX Sham vs. VH SWI, AMX Sham vs. AMX SWI: *p* < 0.0001, VH SWI vs. AMX SWI: *p* = 0.0006). Remarkably, TMEM 119^+^/CD11c^+^ microglia (almost undetectable in sham animals), represented only a small fraction of total microglial cells in SWI-injured mice but were more than fourfold more abundant in SWI mice treated with AMX (Figure 2; VH Sham vs. VH SWI: *p* = 0.0216, VH Sham vs. AMX SWI: *p* < 0.0001, AMX Sham vs. VH SWI: *p* = 0.0287, AMX Sham vs. AMX SWI: *p* < 0.0001, VH SWI vs. AMX SWI: *p* < 0.0001).

Furthermore, we detected a massive (almost eightfold) increase in the number of TMEM119^+^/CD169^+^ cells in mice subject to SWI and treated with AMX (compared to SWI mice treated with vehicle; Figure 2; VH Sham vs. AMX SWI: *p* < 0.0001, AMX Sham vs. AMX SWI: *p* < 0.0001, VH SWI vs. AMX SWI: *p* < 0.0001). Interestingly, almost all TMEM119^+^/CD169^+^ cells (representing about one-third of the total TMEM119^+^ population) were also CD11c^+^ (although the opposite was not true, and almost one-half of TMEM119^+^/CD11c^+^ cells were CD169-), suggesting that a distinct TMEM119^+^ /CD11c^+^/CD169^+^ phenotype was induced by AMX treatment.

**FIGURE 3:**
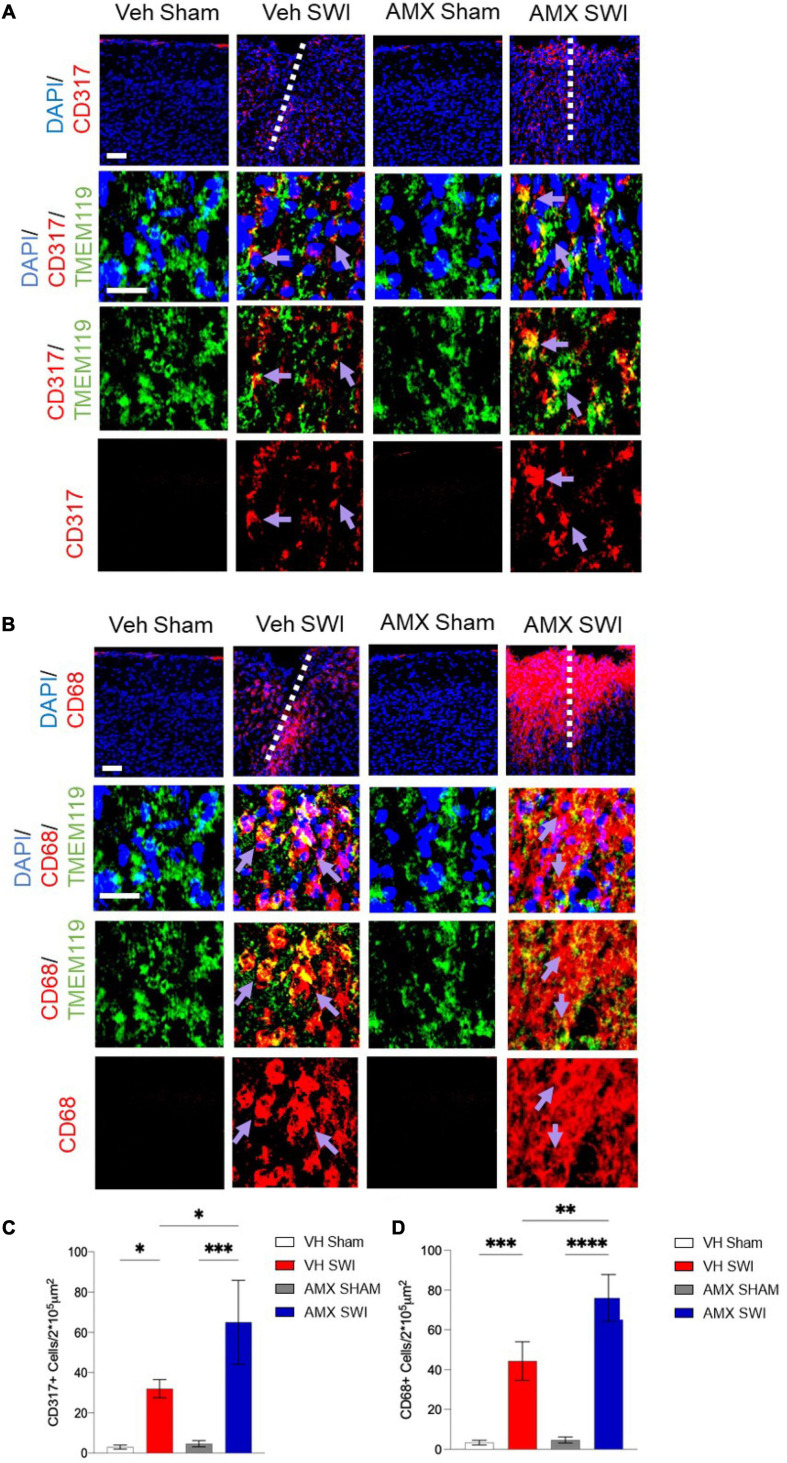
Tbk1 blockade enhances the induction of CD317^+^ interferon-stimulated microglia and CD68^+^ phagocytic microglia. **(A,C)** AMX treatment substantially increased the number of microglia TMEM119^+^ that expressed the interferon-induced CD317 marker upon SWI (at 7 dpi). **(B,D)** AMX upregulated the expression of the phagocytosis marker CD68 in TMEM119^+^ microglia upon SWI. (*n* = 3 animals/group with 4 sections/animal. Average ± SD; two-way ANOVA with Tukey’s *post hoc*; ^∗^*p* < 0.05, ^∗∗^*p* < 0.01 ^∗∗∗^*p* < 0.001, ^****^P < 0.0001; full statistical report in Supplementary Table 5). Upper panel, low magnification; lower panels, insets at high magnification. Scale bars (equal for both sets of panels) 200 μm.

We further explored the diversity of the microglial response triggered by SWI injury under AMX treatment by taking into consideration the activation marker (Ajami et al., 2018; Olde Heuvel et al., 2019) and interferon-activated (Polyak et al., 2013) gene CD317. CD317 is expressed at a very low level (as detected by immunohistological techniques) in microglia in sham-treated mice or even in vehicle-treated SWI mice (Figure 3A; VH Sham vs. VH SWI: *p* = 0.0421, VH Sham vs. AMX SWI: *p* = 0.0005, VH SWI vs. AMX SWI: *p* = 0.0223, AMX Sham vs. AMX SWI: *p* = 0.0006). However, the density of TMEM119^+^CD317^+^ cells was substantially increased in AMX-SWI mice, indicating the interferon-driven massive activation of microglial cells (Figure 3C). Moreover, a minor fraction of microglial cells in the injury site in vehicle-treated mice were positive for the DAM marker CD68, but their number was significantly larger in AMX-treated SWI mice, representing the majority of TMEM119^+^ cells (Figures 3B,D; VH Sham vs. VH SWI: *p* = 0.0008, VH Sham vs. AMX SWI: *p* < 0.0001, VH SWI vs. AMX Sham: *p* = 0.001, VH SWI vs. AMX SWI: *p* = 0.0044, AMX Sham vs. AMX SWI: *p* < 0.0001).

**FIGURE 4:**
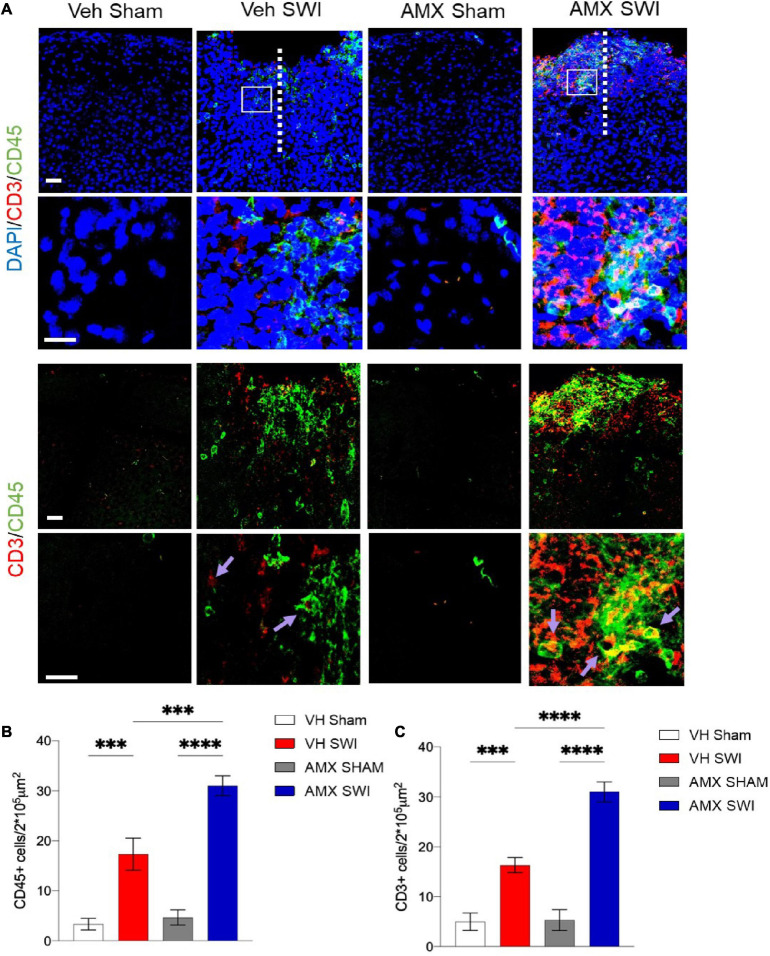
Tbk1 blockade enhances infiltration of the Stab Wound Injury (SWI) by peripheral immune cells. **(A)** In AMX-treated animals (at 7 dpi), the SWI site displayed a substantial increase in the density of CD45-bright cells including peripheral leukocytes; quantified in **(B)** and lymphocytes CD3^+^ cells; quantified in **(C)**; average ± SD; two-way ANOVA with Tukey’s *post hoc* (*n* = 3 animals/group with 4 sections/animal. ^∗∗∗^*p* < 0.001, ^****^p < 0.0001; full statistical report in Supplementary Table 6). Upper panel, low magnification; insets at high magnification. Scale bars (both sets of panels) 200 μm.

Finally, AMX treatment upon SWI also resulted in a significant increase (compared to SWI followed by vehicle administration) in TMEM119-/CD11c^+^ or TMEM119-/CD169^+^ cells, indicating that, besides the massive expansion of DAM-like microglia and triple-positive microglia, AMX treatment also resulted in a substantial increase in recruitment of peripheral immune cells such as dendritic cells and monocytes/macrophages (Figure 2). We further explored this aspect by assessing the number of peripheral leukocytes and the number of lymphocytes infiltrating the site of injury. In line with the CD169 and CD11c data, we found that the SWI contained very few CD45^+^ bright cells at 7 dpi (Figures 4A,B; VH Sham vs. VH SWI, *p* = 0.0002, VH Sham vs. AMX SWI: *p* < 0.0001, VH SWI vs. AMX Sham: *p* = 0.0004, VH SWI vs. AMX SWI: *p* = 0.0002, AMX Sham vs. AMX SWI: *p* < 0.0001), and almost no CD3^+^ lymphocyte in vehicle-treated mice (Figures 4A,C; VH Sham vs. VH SWI, *p* = 0.0003, VH Sham vs. AMX SWI: *p* < 0.0001, VH SWI vs. AMX Sham: *p* = 0.0004, VH SW vs. AMX SWI: *p* < 0.0001, AMX Sham vs. AMX SWI: *p* < 0.0001) (sham mice displayed almost no CD45^+^ or CD3^+^ cell, irrespective of treatment), but their number was substantially higher in the injury site of AMX-treated mice (Figure 4).

### Tbk1 Inhibition by AMX Induces a Disease-Associated Microglia-Like Transcriptional Profile Upon SWI

**FIGURE 5:**
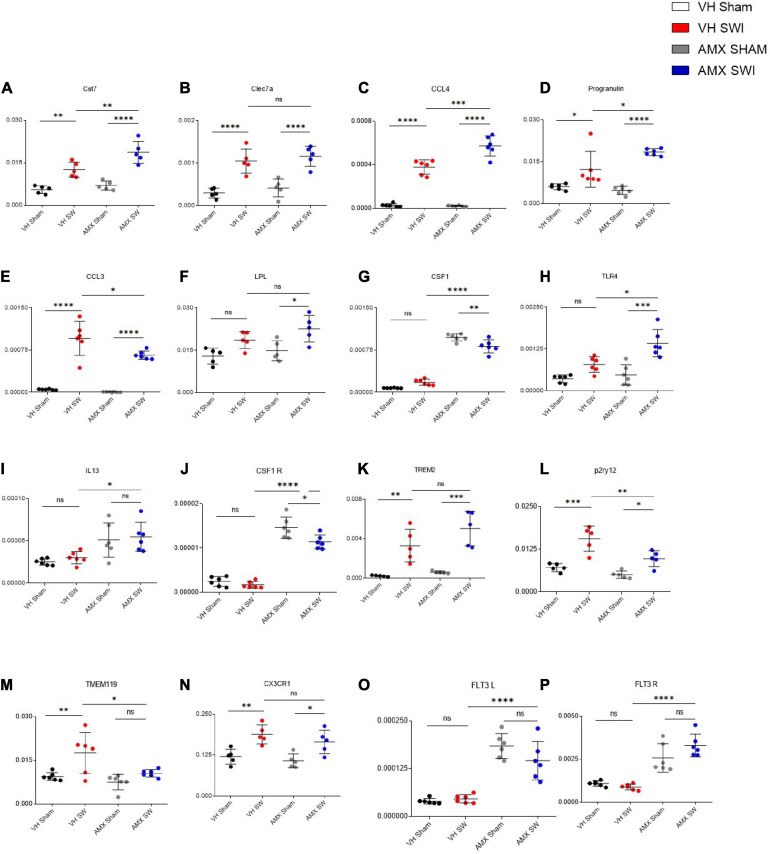
Tbk1 blockade enhances the expression of genes associated with the Disease Associated Microglia (DAM) microglial phenotype after SWI. qPCR from whole-biopsy homogenates were obtained from the injury site (or the corresponding anatomical location in sham mice) from animals treated with either vehicle or AMX at 7 dpi. Among the DAM signature genes, upon SWI, AMX upregulated the expression of Cst7 **(A)**, CCL4 **(C)**, Progranulin **(D)**, CCL3 **(E)**, CSF1 **(G)**, TLR4 **(H)**, and IL-13 **(I)**. Among the genes characteristically downregulated in DAM, AMX substantially reduced the expression of P2ry12 **(L)** and TMEM119 **(M)**. Notably, AMX alone, with or without SWI, upregulated the expression of CSF1 **(G)**, CSF1R **(J)**, Flt3 **(O)** and Flt3R **(P)**. No difference in expression levels was observed upon SWI with or without inhibitor treatment in Clec7a **(B)**, LPL **(F)**, TREM2 **(K)**, and CX3CR1 **(N)**. (*n* = 3 animals/group with 4 sections/animal. Average ± SD, plus individual data points indicated; one-way ANOVA with Tukey post hoc; ns: not significant, ^∗^*p* < 0.05, ^∗∗^*p* < 0.01, ^∗∗∗^*p* < 0.001, ^****^p < 0.0001; full statistical report in Supplementary Table 7).

To confirm and expand the characterization of the microglia phenotype observed upon SWI and AMX treatment, we performed targeted transcriptional profiling of the cortical samples at 7 dpi. First, we considered a set of genes characteristically upregulated (Cst7; Lpl; Clec7a; Ccl3; Ccl4; Prgn; Csf1; Tlr4; Il13; Trem2) or downregulated (Tmem119; P2ry12; Cx3cl1; Csf1r) in DAM (Keren-Shaul et al., 2017; Krasemann et al., 2017). Notably, the expression of the DAM-associated genes Cst7 (Figure 5A; VH Sham vs. VH SWI: *p* = 0.0026), Clec7a (Figure 5B; VH Sham vs. VH SWI: *p* = 0.0003), Ccl4 (Figure 5C; VH Sham vs. VH SWI: *p* < 0.0001), Prgn (Figure 5D; VH Sham vs. VH SWI: *p* = 0.023), and Ccl3 (Figure 5E; VH Sham vs. VH SWI: *p* < 0.0001) was modestly upregulated by SWI in vehicle-treated mice (compared to sham controls). In AMX treated mice, expression of Cst7 (Figure 5A; VH SWI vs. AMX SWI: *p* = 0.0026), Lpl (Figure 5F; AMX SWI vs. AMX SWI: *p* = 0.0164), Ccl4 (Figure 5C; AMX SWI vs. AMX SWI: *p* = 0.0003), Prgn (Figure 5D; VH SWI vs. AMX SWI: *p* = 0.0232), Csf1 (Figure 5G; VH SWI vs. AMX SWI: *p* < 0.0001), Tlr4 (Figure 5H; VH SWI vs. AMX SWI: *p* = 0.0049), Il13 (Figure 5I; VH SWI vs. AMX SWI: *p* = 0.0283), and Csf1r (Figure 5J; VH SWI vs. AMX SWI: *p* < 0.0001) was substantially upregulated, and trends toward upregulation were seen for Trem2 (Figure 5K; AMX SWI vs. AMX SWI: *p* = 0.0001) (whereas Clec7a was not affected by AMX). Conversely, P2ry12 (Figure 5L; VH SWI vs. AMX SWI: *p* = 0.0056) and Tmem119 (Figure 5M; VH SWI vs. AMX SWI: *p* = 0.0421) were significantly downregulated and Cx3cr1 (Figure 5N; AMX SWI vs. AMX SWI: *p* = 0.0217) showed a trend toward downregulation (whereas Csf1r was upregulated). Thus, the genes up- and down-regulated by AMX treatment showed a strong resemblance with the expression fingerprint of DAM microglia, supporting the hypothesis that AMX enhances the induction of DAM-like cells upon SWI (since the transcriptional profile is not completely matching the DAM genome-wide characterization, we opt for the “DAM-like” definition). Of note, TMEM119 mRNA was downregulated in AMX-treated SWI mice but immunoreactivity for TMEM119 was still readily detectable (Figure 2), in agreement with previous observations of persisting TMEM119 proteins when mRNA levels are reduced (Satoh et al., 2016; Krasemann et al., 2017).

Upon closer inspection, we noticed that a subset of genes (Il13, Csf1, Csf1r) was upregulated by AMX alone (in AMX-treated sham-operated mice, compared to vehicle-treated mice subject to either sham or SWI); actually, expression of Csf1r (Figure 5J) and Csf1 (Figure 5G) (inducers of CD11c^+^ microglia; Wlodarczyk et al., 2019) was slightly decreased by AMX (while still being substantially higher than in vehicle controls). We further explored this apparent “priming” effect of AMX alone by assessing the expression of Flt3 (Figure 5O) and Flt3r (Figure 5P) (characteristic of CD11c^+^ microglia (Immig et al., 2015). Most notably, AMX increased the expression of both Flt3 and Flt3r both in sham and SWI mice (compared to vehicle control), with the little additional effect of the injury.

Thus, Tbk1/IKK-ε blockade in SWI is associated with the upregulation of DAM-like transcriptional profiles (in agreement with the histological findings). Furthermore, Tbk1 blockade alone is sufficient to upregulate a few, but not all, genes associated with CD11c^+^ microglia.

### Tbk1/IKK-ε Inhibition Upregulates Inflammatory Cytokines but Downregulates Chemokines in the Cortex Subject to SWI

**FIGURE 6:**
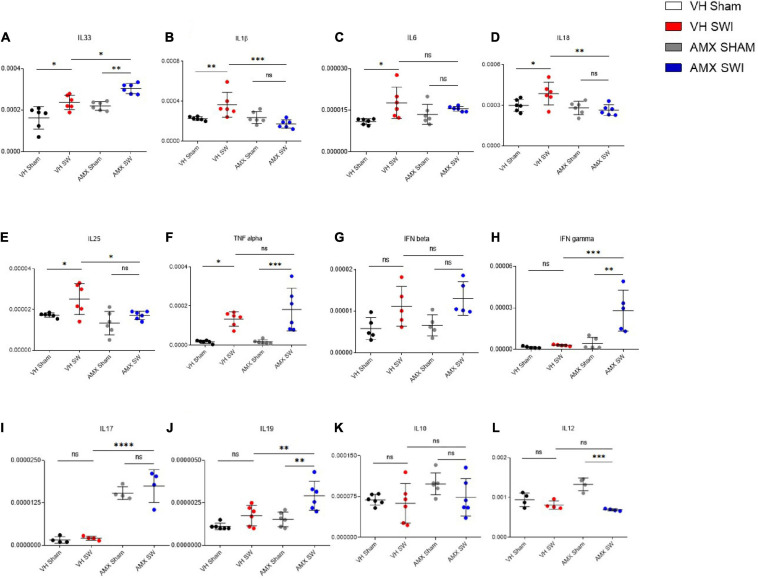
Tbk1 blockade induces a distinct inflammatory cytokines profile upon Stab Wound Injury (SWI). qPCR from whole-biopsy homogenates were obtained from the injury site (or the corresponding anatomical location in sham mice) from animals treated with either vehicle or AMX at 7 dpi. AMX treatment after SWI significantly upregulated the expression of IL-33 **(A)**, IFN-γ **(H)**, IL-17 **(I)** and IL-19 **(J)**. Interestingly, AMX after SWI (7 dpi) also significantly reduced the expression of IL-1β **(B)**, IL-18 **(D)**, IL-25 **(E)**. Notably, AMX alone, irrespective of concomitant SWI, substantially increased the levels of IL-17 mRNA **(I)**. No difference in expression levels was observed upon SWI with or without inhibitor treatment in IL6 **(C)**, TNF alpha **(F)**, IFN beta **(G)**, IL10 **(K)**, and IL12 **(L)**. (*n* = 3 animals/group with 4 sections/animal. Average ± SD, plus individual data points indicated; two-way ANOVA with Tukey post hoc; ns: not significant, ^∗^*p* < 0.05, ^∗∗^*p* < 0.01, ^∗∗∗^*p* < 0.001, ^****^p < 0.0001; full statistical report in Supplementary Table 8).

Having observed the massive upregulation of CD11c^+^ microglia density and its gene expression profile, as well as the enhanced leukocytes and lymphocytes infiltration, in the injured cortex of mice treated with AMX, we further explored if these corresponded to a distinct neuroimmunological profile. To this aim, we assessed the expression of 12 inflammatory and immunomodulatory cytokines (Il-1b, Tnf-a, Il-33, Ifn-b, Il-6, Il-17, Il-12, Ifn-g, Il-18, Il-10, Il-19, Il-25) in cortical samples obtained at 7 dpi from mice treated with vehicle or AMX and subject to sham surgery or SWI (as above). Interestingly, AMX treated significantly upregulated Il-33 (Figure 6A; AMX Sham vs. AMX SWI: *p* = 0.0034) expression, whereas Il-1b (Figure 6B), Il-6 (Figure 6C), Il-18 (Figure 6D), and Il-25 (Figure 6E) were downregulated. Meanwhile, Tnf-a (Figure 6F; VH Sham vs. VH SWI: *p* = 0.0119), Il-6 (Figure 6C; VH Sham vs. VH SWI: *p* = 0.0134) (and, as trend only, Ifn-b; Figure 6G) were upregulated by SWI but unaffected by AMX. Most notably, when AMX was administered to SWI mice, it caused the massive elevation in lymphocyte-specific cytokines, such as Ifn-g (Figure 6H; VH SWI vs. AMX SWI: *p* = 0.0005), Il-17 (Figure 6I; VH SWI vs. AMX SWI: *p* < 0.0001), and Il-19 (Figure 6J; VH SWI vs. AMX SWI: *p* = 0.0102) (only trends were observed for Il-10; Figure 6K). Upon closer inspection, once again we detected a significant effect of AMX alone in elevating Il-17 (Figure 6I) and Il-12 (Figure 6L) expressions already in absence of SWI (Il-12 was then actually downregulated by SWI).

**FIGURE 7:**
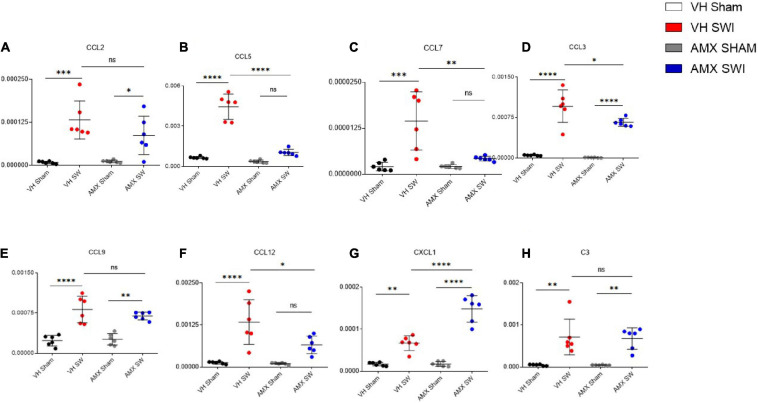
Tbk1 blockade modifies the chemokine profile after Stab Wound Injury (SWI) injury. qPCR from whole-biopsy homogenates were obtained from the injury site (or the corresponding anatomical location in sham mice) from animals treated with either vehicle or AMX at 7 dpi. AMX strongly downregulated the expression of CCL-5 **(B)**, CCL-7 **(C)**, CCL-3 **(D)**, CCL-3 **(E)**, CCL-12 **(F)** but not of CCL-2 **(A)**, and strongly upregulated the expression of CXCL1 **(G)**. Expression of the complement factor C3 was unchanged **(H)**. (*n* = 3 animals/group with 4 sections/animal. Average ± SD, plus individual data points indicated; two-way ANOVA with Tukey post hoc; ns: not significant, ^∗^*p* < 0.05, ^∗∗^*p* < 0.01, ^∗∗∗^*p* < 0.001, ^****^p < 0.0001; full statistical report in Supplementary Table 9).

Finally, we explored if the altered cytokine profile, including pro- and anti-inflammatory changes, would affect the landscape of chemokines involved in regulating inflammation and chemotaxis. We assessed the expression of the C-C chemokines Ccl2 (Figure 7A), Ccl5 (Figure 7B), Ccl7 (Figure 7C), Ccl3 (Figure 7D), Ccl9 (Figure 7E), Ccl12 (Figure 7F) as well as the C-X-C chemokine Cxcl1 (Figure 7G) and the complement factor C3 (Figure 7H). Whereas all these mediators were upregulated by SWI (in vehicle-treated mice), AMX treatment exerted a mainly suppressive effect, resulting in the downregulation of Ccl3 (Figure 7D; VH SWI vs. AMX SWI: *p* = 0.0162), Ccl7 (Figure 7C; VH SWI vs. AMX SWI: *p* = 0.0014), Ccl12 (Figure 7F), and Ccl5 (Figure 7B; VH SWI vs. AMX SWI: *p* < 0.0001) (as a trend, also for Ccl9 and Ccl2; Figure 7). Surprisingly, Cxcl1 was strongly upregulated by AMX in SWI mice (Figure 7G) (C3 expression remained unaffected by AMX; Figure 7H). Thus, despite a substantial increase in inflammatory cells and microglia and the upregulation of inflammatory cytokines, AMX largely suppresses chemokine induction with the notable exception of Cxcl1 (which rather corresponds to the upregulation of Il-17).

### The Expansion of CD11c^+^ Microglia Is Reversed by AMX Withdrawal

**FIGURE 8:**
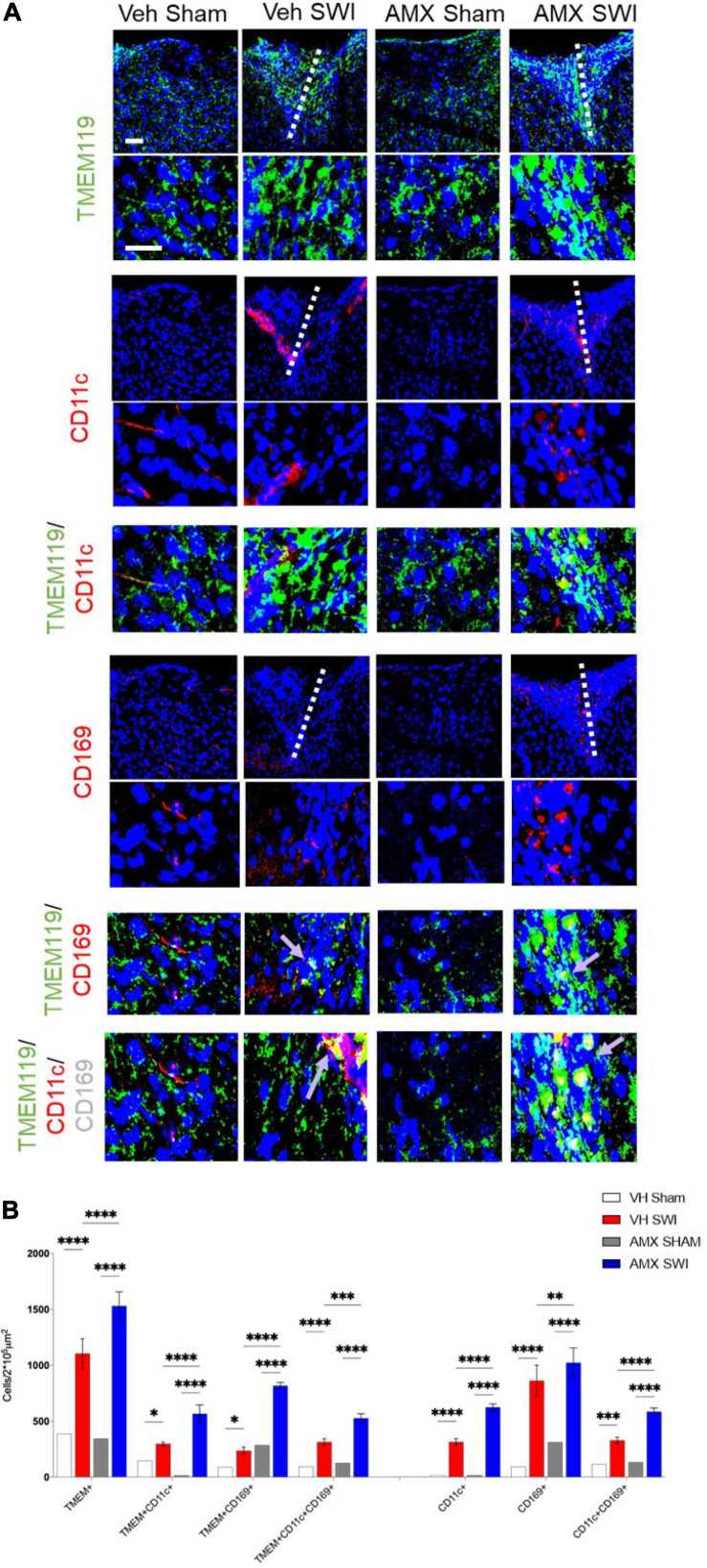
Persistent effect of 7-days Tbk1 blockade on microglial DAM-like phenotype after 33 days of washout. **(A,B)** Mice were subject to cortical SWI injury and treated with AMX for 7 days; AMX was then withdrawn and mice were sacrificed 43 days later (40 days after injury). In AMX-treated mice, even after long wash-out, the density of CD11c^+^ and CD169^+^ microglia (TMEM119^+^) remained significantly higher than in vehicle-treated animals. (*n* = 3 animals/group with 4 sections/animal. Average ± SD, plus individual data points indicated; two-way ANOVA with Tukey *post hoc*; ns: not significant, ^∗^*p* < 0.05, ^∗∗^*p* < 0.01, ^∗∗∗^*p* < 0.001, ^****^P < 0.0001; full statistical report in Supplementary Table 10). Length of scale bars for both low-magnification and high-magnification insets is 200 μm.

Next, we explored if the AMX-induced altered expansion of CD11c^+^ and CD169^+^ microglial subpopulations would result in a persistent modification of the microglial populations. To this aim, we subjected mice to SWI (or sham), treated them with AMX or vehicle for 7 days but we sacrificed the mice at 40 days, after 33 days of AMX wash-out. We observed that microglial density was still significantly higher in the injury site of AMX-treated mice compared to vehicle-treated ones (Figure 8A; VH SWI vs. AMX SWI: *p* = 0.0025). However, the density of TMEM119^+^ /Cd11c^+^ cells and TMEM119^+^/CD11c^+^/CD169^+^ cells was still higher than in vehicle treated mice, but showed a lower absolute value than at the 7 days timepoint (Figure 8B; TMEM119/CD11c: VH SWI vs. AMX SWI: *p* = 0.0003, TMEM119/CD169: VH SWI vs. AMX SWI: *p* < 0.0001). A similar situation was observed when the AMX treatment was prolonged for 21 days and the mice were sacrificed 20 days after the last dose. Thus, although the expansion of the microglial population caused by the AMX treatment may be persistent, the CD11c^+^ and CD11c/CD169^+^ phenotype may slowly vanish upon AMX withdrawal.

### Treatment With Tbk1/IKK-ε Inhibitor Results in Increased Astrogliosis, Chondroitin-Sulfate Proteoglycans Deposition and Loss of Post-synaptic Proteins After SWI

**FIGURE 9:**
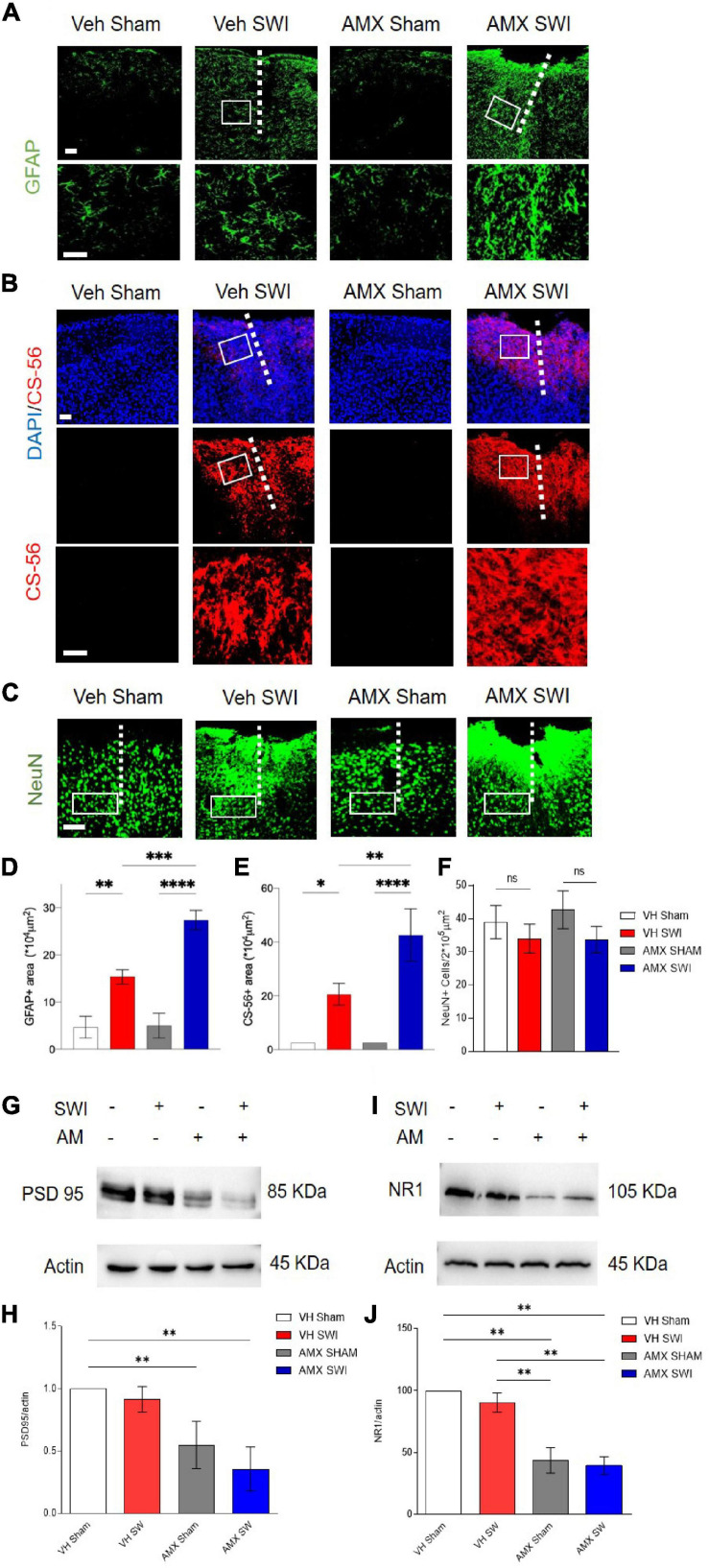
Tbk1 blockade enlarges the gliotic scar and amplifies the deposition of CPSG at 7dpi after Stab Wound Injury (SWI). **(A,D)** The density of reactive astrocytes (GFAP^+^) is substantially larger at 7 dpi in mice treated with AMX for 7 days after SWI compared to vehicle-treated controls. **(B,E)** The extent of the CSPG scar deposition (as revealed by immunostaining with the CS-56 antibody) is substantially increased by the treatment with AMX for 7 days after SWI injury. **(C,F)** The density of NeuN^+^ cells is unaffected by either vehicle or AMX treatment. Western blots of cortical biopsies (at 7 dpi) homogenates show decreased levels of **(G,H)** PSD-95 and **(I,J)** NR1. (*n* = 3 animals/group with 4 sections/animal. *n* = 4 animals for the vehicle treated group and *n* = 6 animals for the amlexanox treated group. Average ± SD; one-way ANOVA with Tukey *post hoc*; ns: not significant, ^∗^*p* < 0.05, ^∗∗^*p* < 0.01, ^∗∗∗^*p* < 0.001, ^****^P < 0.0001; original uncropped blots in Supplementary File 2; full statistical report in Supplementary Table 11). Length of scale bars for both low-magnification and high-magnification insets is 200 μm.

Finally, we explored the consequences of the strongly enhanced DAM-like phenotype and lymphocyte infiltration caused by AMX in mice subject to SWI. First, we considered the extent of astroglial scarring (Förstner et al., 2018; Frik et al., 2018). We found that SWI induced a substantial increase in GFAP^+^ astrocytes in and around the lesion site (Frik et al., 2018) at 7dpi, but also that astrogliosis was significantly increased in AMX-treated mice (Figures 9A,D; VH SWI vs. AMX SWI: *p* = 0.0007). Likewise, the deposition of glycosaminoglycans such as in chondroitin sulfate proteoglycans (CSPG; as detected the immunostaining with CS56 antibody) was massively expanded in the lesion site of AMX-treated mice (Figures 9B,E; VH SWI vs. AMX SWI: *p* = 0.0041). Second, we considered the consequences of the DAM-like induction on neuronal and synaptic phenotypes. The number of NeuN^+^ (neurons) cells per area unit was not significantly affected by the SWI, either in vehicle or in AMX-treated mice (Figures 9C,F). On the contrary, the amount of the marker of excitatory postsynaptic structures PSD-95 (assessed by WD in whole-cortical biopsy protein extract) decrease in AMX-sham but showed a trend toward being further decreased in AMX SWI samples (Figures 9G,H; VH Sham vs. AMX Sham: *p* = 0.0018, VH SWI vs. AMX SWI: *p* = 0.0086). Likewise, the NMDAR glutamate receptor subunit NR1 was reduced by AMX alone as well as in AMX-SWI samples (Figures 9I,J; VH Sham vs. AMX Sham: *p* = 0.0028, VH SWI vs. AMX SWI: *p* = 0.0052).

Thus, AMX treatment results in a substantial expansion of astroglial and CSPG scar and a significant loss of synaptic proteins, indicating an ultimately detrimental consequence of the enhanced microglial inflammation and leukocyte infiltration.

## Discussion

Our findings show that acute blockade of Tbk1/IKK-ε using the small molecule AMX determines a substantial shift in the neuroinflammation associated with SWI. We detected a massive enhancement in the number and reactivity of microglial cells with the appearance of large numbers of microglial cells with phenotype (defined DAM-like) strongly resembling the DAM described in Alzheimer’s disease and other aging-associated neurodegenerative conditions. Furthermore, we observed a substantial increase in the infiltration of peripheral lymphocytes and leukocytes, associated with a cytokine and chemokine profile dominated by the upregulation of IL-17, IFN-gamma, and CXCL1. Interestingly, AMX alone, without the application of SWI, appeared to prime the immune response toward increased reactivity, upregulating the levels of IL-13, IL-17, CSF1/CSF1R, and Flt3/Flt3R. The ultimate impact of this increased activation of microglia with DAM-like phenotype and lymphocyte infiltration appears to be detrimental since it corresponded to a substantial expansion of astrocytic and G scar. Thus, Tbk1/IKK-ε inhibition appears to lead to an increased inflammatory response with enlarged astroglial scarring. It must be underscored that the SWI model is especially suited to study glial responses to injury and does not recapitulate the complexity of other TBI conditions, such as blunt trauma with or without hematoma and skull fracture.

In terms of molecular mechanisms, the dual inhibition Tbk1 and IKK-ε by AMX makes it not possible to disentangle the contribution of either kinase. Nevertheless, both kinases display a certain degree of convergence in terms of signaling cascades: AMX treatment has been shown to result in a decrease in canonical NF-kB signaling (Cheng C. et al., 2018; Möser et al., 2019) as well as STING-dependent NF-kB activation (Balka et al., 2020). Furthermore, Tbk1 and IKK-ε are critical components of the Interferon signaling cascade (Fitzgerald et al., 2003) as well as in limiting the phosphorylation and the signaling through RIPK1 signaling (Lafont et al., 2018). Elucidation of any non-redundant role of each kinase in the phenotype evoked by AMX is the object of active investigation.

**FIGURE 10:**
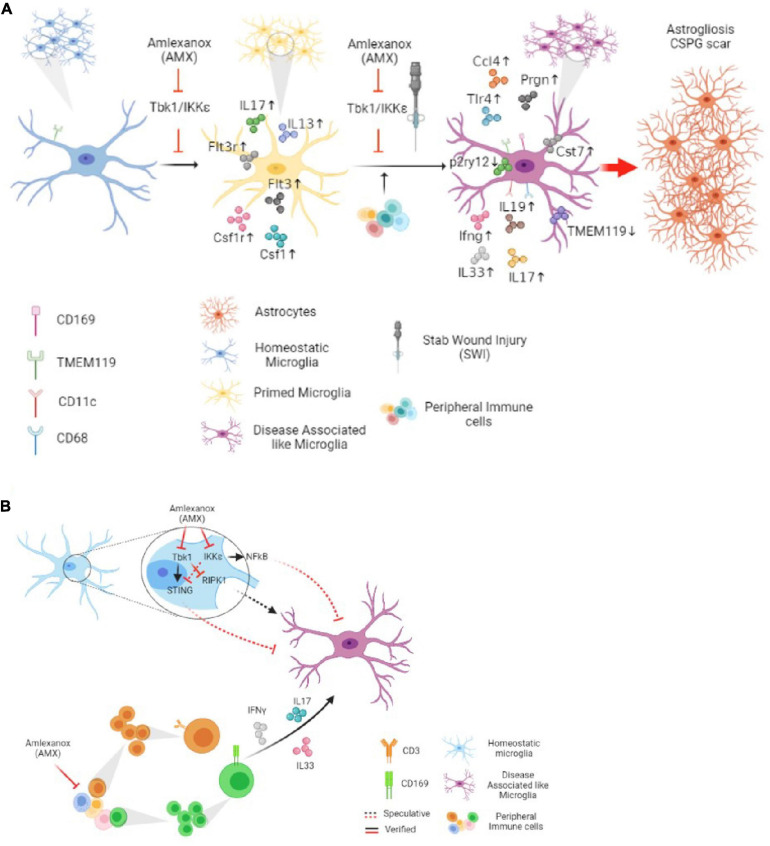
Tbk1 inhibition by Amlexanox modifiedy the microglial and immune response in cortical Stab Wound Injury (SWI) injury. **(A)** Blockade of Tbk1 alone by Amlexanox (AMX), in absence of additional damage, is sufficient to increase the expression of IL-17, CSF1, CSF1R, Flt3, Flt3R, and IL-13, indicating an initial baseline pro-inflammatory state; upon SWI injury, AMX administration causes the substantial expansion of reactive microglia with immunohistochemical and expression profile similar to the Disease-Associated Microglia described in neurodegenerative conditions as well as the significant increase in the infiltration by lymphocytes and monocytes/macrophages. The ultimate effect appears to be increased astrogliosis and extensive deposition of CSPG scar. **(B)** At least two molecular mechanisms are proposed for AMX effect on microglial phenotype: the de-repression of the RIPK1 pathway and the de-activation of the STING pathway. The concomitant upregulation of inflammatory cytokines, some of lymphocytic origin such as IL-17 and IFN-γ, is likely to contribute to the peculiar microglial phenotype.

In terms of cellular mechanisms and interactions, AMX displayed at least two remarkable effects in the SWI model which may be implicated, possibly in cooperation, in this overall detrimental effect: increased lymphocytes and monocytes infiltration and, most notably, the induction of a DAM-like (Tmem119^+^CD11c^+^ as well as the Tmem119^+^CD11c^+^CD169^+^) phenotype in local microglia. Both effects seem to point toward an enhancement of the reaction to the injury, in contrast to the anticipated anti-inflammatory effects of AMX and of the inhibition of Tbk1 and IKK-ε (Figure 10A).

Which mechanisms may link the blockade of Tbk1 and IKK-ε to the increased infiltration of lymphocytes in the injury site? Loss of Tbk1 alone has been previously associated with a reduced threshold for lymphocytes activation, the generation of Th1 and Th17 subpopulations, and with the substantial enhancement of IFN-gamma secretion (Yu et al., 2015), although, at the same time, retention of lymphocytes in lymph nodes and reduced brain inflammation was observed upon EAE induction in mice lacking Tbk1 in lymphocytes (Yu et al., 2015). Notably, AMX administration in the context of EAE also results in a decreased activation of Th1 and Th17 and in the reduction of their infiltration of the brain (Quan et al., 2019). In contrast, we do observe an increase in lymphocyte trafficking in the CNS upon AMX treatment, suggesting that the outcome of Tbk1/IKK-ε inhibition may be dependent on the type of injury and the timing of administration (e.g., before injury or after). In fact, partial deletion of Tbk1 alone is associated with enhanced inflammatory profile in aging (Bruno et al., 2020) but reduces the microglial activation associated with neurodegeneration (Brenner et al., 2019). Furthermore, several of the cytokines upregulated upon AMX treatment may be involved in the increased leukocytes and lymphocytes infiltration. In fact, the induction of IL-17 may critically contribute to the recruitment of other leukocytes through the elevation of the chemokine Cxcl1 (Wojkowska et al., 2017). Additional cytokines upregulated by AMX, such as IL-13 and IFN-γ appear to be able to enhance the secretion of IL-6 and TNF-α as well as the release of Nitric Oxide from microglia (Quarta et al., 2021). We also identified a distinctive elevation in IL-19 mRNA. This cytokine is known to be predominantly secreted by microglia subject to inflammatory stimuli (such as LPS), and to act back on microglia itself to reduce the induction of inflammatory cytokines such as suppress IL-6 and TNF-α; it is therefore thought to be induced by inflammation as part of a negative feedback loop (Horiuchi et al., 2015).

One of the most striking effects of AMX treatment in SWI is the enhancement of microglial (TMEM119^+^) subpopulations highly expressing CD11c (TMEM119^+^CD11c^+^), or CD169 (TMEM119^+^CD169^+^) and the double-positive TMEM119^+^CD11c^+^CD169^+^ subpopulation. These subpopulations were barely detected in vehicle-treated SWI samples but were substantially represented in the AMX treated mice. TMEM119^+^ CD11c^+^ microglia (which may encompass more than one subset) has been recently identified as DAM, a subpopulation characteristically associated with neurodegenerative diseases (Keren-Shaul et al., 2017; Krasemann et al., 2017; Cao et al., 2021) and white matter aging (Sato-Hashimoto et al., 2019; Safaiyan et al., 2021). Expression profiling of DAM has revealed the upregulation of genes involved in lipid metabolism (most notably, ApoE) and apparently reduced capacity for engulfment, phagocytosis and degradation (Krasemann et al., 2017; Ulland et al., 2017). Although often associated with degenerative processes (Deczkowska et al., 2018), it has been reported to be involved in either protective processes (such as amyloid plaque compaction in AD, debris clearing in stroke or myelin damage; Cignarella et al., 2020; Meilandt et al., 2020; Lee et al., 2021) or detrimental processes (Götzl et al., 2019) depending on the pathological condition. On the other hand, CD169 expression characterizes actively phagocytosing cells (O’Neill et al., 2013); CD169 is normally not expressed in microglia but a distinct population of CD169^+^ microglia has been associated with active phagocytosis of myelin and it is highly pathogenic in EAE models (Bogie et al., 2018; Ostendorf et al., 2021). Together the TMEM119^+^ CD169^+^ and the currently uncharacterized population of TMEM119^+^ CD11c^+^ CD169^+^ are anticipated to be associated with massive phagocytosis of neurons and myelin in the site of injury (Figures 10A,B).

How does the inhibition of Tbk1 and IKK-ε lead to induction of a DAM-like phenotype in microglia? Several non-mutually exclusive molecular pathways may be involved. Recent evidence suggests a model in which Tbk1 may suppress or limit the appearance of a DAM-like phenotype in microglia in a cell-autonomous way (i.e., independently of the increased leukocytes infiltration). The DAM-like phenotype is dependent on the activity of the RIPK1, and genetic or pharmacological inhibition of RIPK1 prevents the upregulation of the DAM marker Cst7 and reverses the compromised phagocytic/autophagic degradation pathway (Ofengeim et al., 2017). Indeed, Tbk1 is one of the most important upstream inhibitors of RIPK1, and phosphorylation of RIPK1 by Tbk1 on T189/T190 prevents RIPK1 activation and genetic or pharmacological inhibition of Tbk1 results in an enhanced RIPK1 pathway activation and microglial reactivity (Xu et al., 2018). Moreover, both Tbk1 and IKK-ε are able to phosphorylate Ik-B (leading to its degradation) and contribute to the activation of NF-kB-dependent transcriptional responses so that blockade of the two kinases would result in a decrease in canonical activation of NF-kB in immune cells (Carr et al., 2020); at the same time, upregulation of non-canonical NF-kB signaling has been reported upon the Tbk1 blockade (Jin et al., 2012). Interestingly, loss of the p50 subunit of NF-kB is associated with increased reactive microglial morphology and enhanced secretion of TNF-α upon LPS challenge in aged mice (Taetzsch et al., 2019), suggesting that reduced canonical activation of NF-kB (like in case of AMX treatment) may also translate into increased microglial reactivity. Third, a subset of microglia in AMX-treated SWI mice display high levels of CD317, a well-known type-I IFN-induced gene (Polyak et al., 2013) suggesting the possibility that AMX may drive the induction of an interferon-response microglial phenotype (Sala Frigerio et al., 2019); however, these cells are relatively low in absolute number and the levels of IFNb mRNA were not actually modified. On the other hand, Tbk1 phosphorylates STING and Tbk1 and IKK-ε are essential for its signaling through IRF3 and NF-kB (Balka et al., 2020; Yum et al., 2021); in our experimental conditions, we verified that AMX treatment reduced the levels of STING phosphorylation. Recently it has been shown that activation of STING signaling pathway in microglia is actually responsible for a decrease in microglial reactivity (Mathur et al., 2017); furthermore, microglia can undergo STING-dependent apoptosis to limit IFN production during viral infections (Reinert et al., 2021). Therefore, loss of Tbk1/IKK-ε-STING may actually contribute to the enhanced microglial reactivity and increased accumulation seen upon AMX treatment. Finally, contributions to the induction of the DAM-like phenotype may be provided by infiltrating lymphocytes’cytokines: IL-17 is well-known to induce microglial activation (Chen et al., 2020), although the specific role in the DAM phenotype is not yet clear.

Interestingly, some of the cytokines related to the pro-inflammatory environment due to AMX appear to be elevated already in sham-operated mice, independently of SWI. It is, thus, conceivable that AMX alone may prime the inflammatory state of microglia, with SWI triggering the DAM-like phenotype, in a multi-step process reminiscent of what has been formerly hypothesized in other conditions (Keren-Shaul et al., 2017). Furthermore, the AMX-induced phenotype closely resembles the one observed upon aging in white matter microglia. This effect is also compatible with a role for the Tbk1 pathway in inflammaging (Franceschi et al., 2018) i.e., in driving low-grade baseline inflammatory activation and in amplifying responses upon injury (Xu et al., 2018; Bruno et al., 2020; Gerbino et al., 2020). Although downregulation of TBK1 signaling takes place in aging (Xu et al., 2018), it remains to be seen if acute TBK1 inhibition faithfully mimics the long-term decline in TBK1 activity in aging, and their associated inflammaging phenotypes.

The ultimate role of DAM is currently the object of an ongoing debate: when first identified, DAM was judged to be a beneficial component of the response to AD-associated amyloid plaques, and enhancement of DAM generation was hypothesized to be a viable therapeutic strategy (Keren-Shaul et al., 2017; Deczkowska et al., 2018; Ewers et al., 2020). On the other hand, the kinetics of DAM appearance suggested that it may be a detrimental component of neurodegeneration (Rangaraju et al., 2018). DAM was shown to be involved in the removal of apoptotic neurons (Krasemann et al., 2017), and therefore it would have a beneficial role in tissue injury by removing debris. In the context of acute injury, deletion of TREM2, which is normally involved in the induction of reactive microglia and whose deletion is anticipated to prevent such reactive phenotype (Nugent et al., 2020), has been reported to be beneficial in traumatic brain injury (Saber et al., 2017), implying a detrimental role. A similar effect was reported in stroke models (Wu et al., 2017; Chen et al., 2020). Our data seems to point to a similar detrimental role of DAM-like microglia in SWI since the blockade of Tbk1 and the massive expansion of the CD11c^+^ microglia is associated with increased deposition of CSPG and increased astrogliosis. To date, individual immune peripheral and microglial contributions to this ultimate effect are to be investigated, since one of the limitations of the present study is the use of a systemic inhibitor of Tbk1/IKK-ε.

Although the induction of a DAM-like phenotype by AMX in the context of SWI appears detrimental, as measured by the larger astrocytic scar, the induction of a DAM phenotype as been hypothesized to be beneficial in the context of Alzheimer Disease (AD; Lewcock et al., 2020). In AD, genetic variants reducing TREM2 signaling causes the reduced induction of DAM, which normally surround amyloid plaques and display active phagocytosis and lipid metabolism, with beneficial consequences on synaptic loss, amyloid peptides burden and dystrophic dendrites (Meilandt et al., 2020; Lee et al., 2021). Since TREM2 with reduced signaling appear to be risk factors for AD and are associated with incomplete DAM induction, strategies have been developed to upregulate TREM2 signaling using genetic (e.g., Lee et al., 2018) or antibody (Cignarella et al., 2020; Lewcock et al., 2020) approaches. In this context, our findings suggest that AMX (and more broadly, the Tbk1/IKK-ε inhibition) may contribute to enhancing the induction of DAM. However, increased number of DAM is actually associated with neurodegeneration upon progranulin loss (Götzl et al., 2019), implying that the effect of AMX may be difficult to extrapolate, due to the beneficial or detrimental effects of reactive microglia (Aguzzi et al., 2013) and may be worth exploring in different disease models. It must be noted that the conditions of acute damage with substantial immune cells infiltration are not similar to those of chronic, slowly progressive neurodegenerative conditions: thus, the actual phenotype of microglial cells and their impact on the ongoing pathogenic process may be highly disease- and stage-dependent (e.g., Ouali Alami et al., 2018).

## Data Availability Statement

The raw data supporting the conclusions of this article will be made available by the authors, without undue reservation.

## Ethics Statement

The animal study was reviewed and approved by the Regierungspraesidium Tubingen, no. 1379.

## Author Contributions

FR conceived the study, supervised the project, and planned the experiments. RR collected samples, performed analyses, prepared figures, and worked on the manuscript. LT performed staining, western blot, and PCR experiments. AO performed preliminary analysis for GFAP and CS56 immunostainings. ZL performed expression analysis. DW performed SWI injury and western blot experiments. TB and JK provided critical reagents and resources. FR, JW, TB, JK, and AL contributed reagents and participated in the drafting of the manuscript. FR and RR contributed to the final version of the manuscript. All authors read and approved the manuscript.

## Conflict of Interest

The authors declare that the research was conducted in the absence of any commercial or financial relationships that could be construed as a potential conflict of interest.

## Footnotes

1 https://www.jax.org/strain/100012

## Supplementary Material

Supplementary Table 1 Sequences of the primers used in the qPCR experiments.

Supplementary Table 2 List of materials used in the study.

Supplementary Table 3 Detailed statistics for the experiments depicted in Figure 1.

Supplementary Table 4 Detailed statistics for the experiments depicted in Figure 2.

Supplementary Table 5 Detailed statistics for the experiments depicted in Figure 3.

Supplementary Table 6 Detailed statistics for the experiments depicted in Figure 4.

Supplementary Table 7 Detailed statistics for the experiments depicted in Figure 5.

Supplementary Table 8 Detailed statistics for the experiments depicted in Figure 6.

Supplementary Table 9 Detailed statistics for the experiments depicted in Figure 7.

Supplementary Table 10 Detailed statistics for the experiments depicted in Figure 8.

Supplementary Table 11 Detailed statistics for the experiments depicted in Figure 9.

Supplementary File 1 Uncropped Western Blot from Figure 1.

Supplementary File 2 Uncropped Western Blot from Figure 9.
