# Supplementary figures and images for: Acute TBK1/IKK-ε Inhibition Enhances the Generation of Disease-Associated Microglia-Like Phenotype Upon Cortical Stab-Wound Injury
Source: Front Aging Neurosci. 2021 Jul 13;13:684171. doi: 10.3389/fnagi.2021.684171 (PMC8313992; doi:10.3389/fnagi.2021.684171)

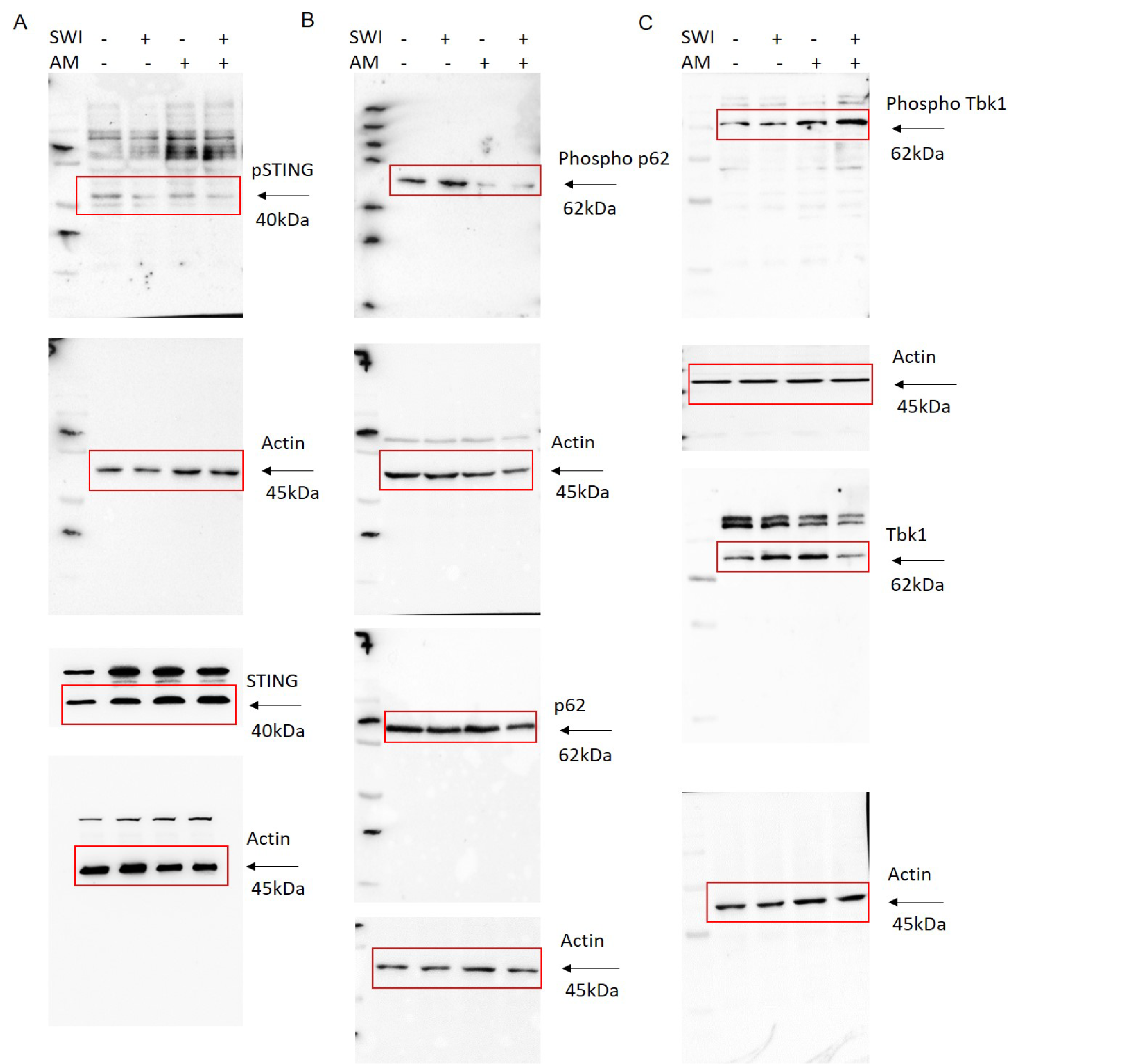

Supplement: Supplementary File 1 — Uncropped Western Blot from Figure 1. [file Image_1.TIF]

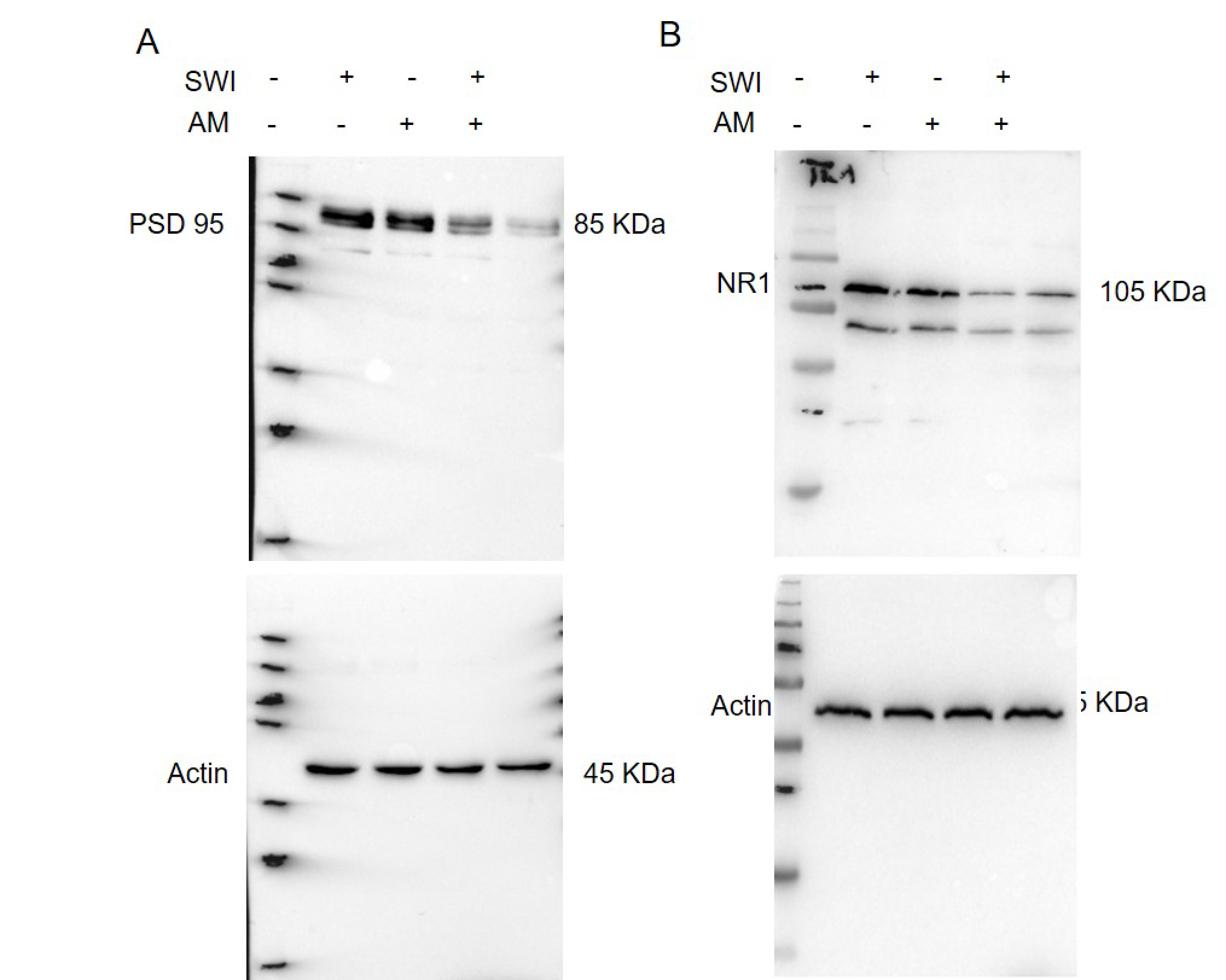

Supplement: Supplementary File 2 — Uncropped Western Blot from Figure 9. [file Image_2.TIF]
